# Supplementary material for: Cytokines and Lymphoid Populations as Potential Biomarkers in Locally and Borderline Pancreatic Adenocarcinoma
Source: Cancers (Basel). 2022 Dec 5;14(23):5993. doi: 10.3390/cancers14235993 (PMC9739487; doi:10.3390/cancers14235993)
Supplement: Supplementary file 1 [file cancers-14-05993-s001.zip › supplementary/Supplementary Figure S1.pdf]

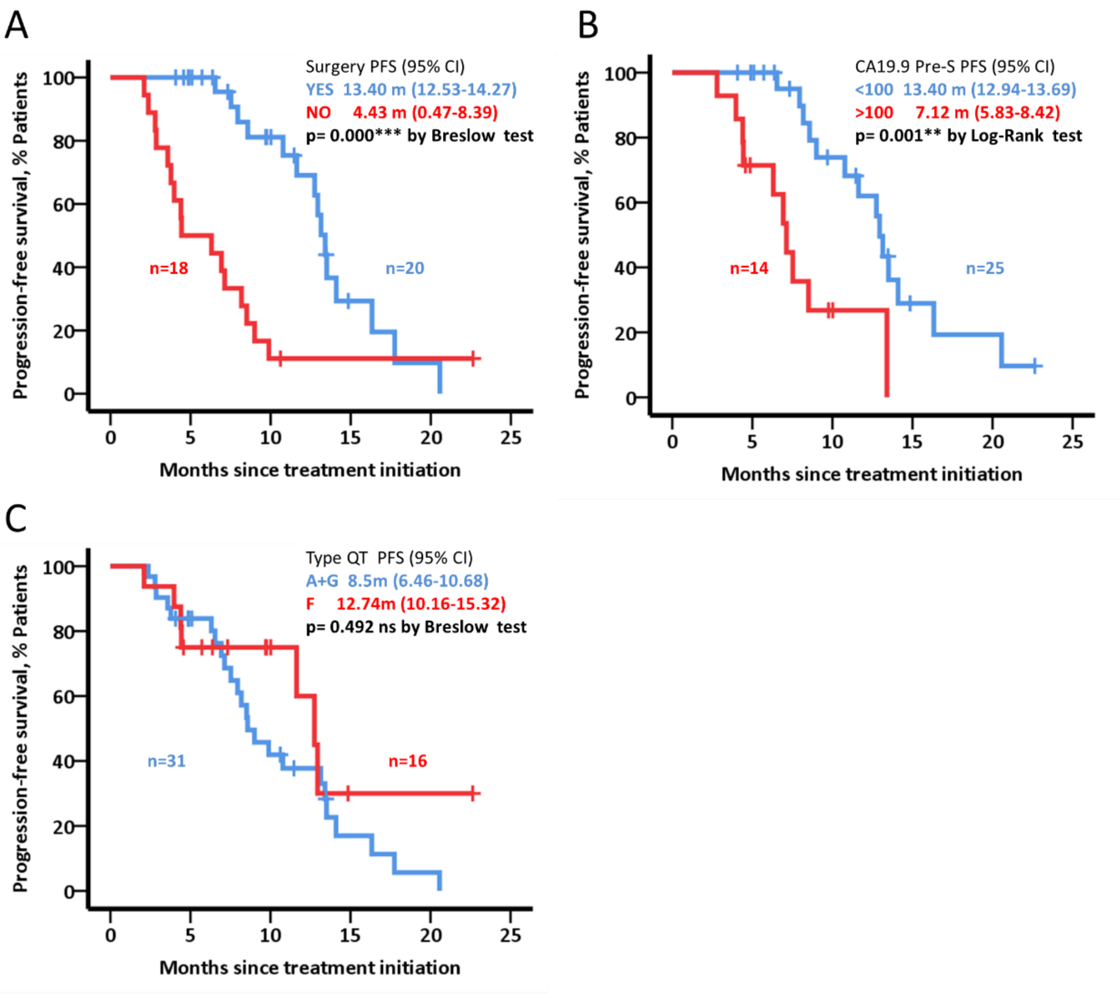

Supplementary Figure S1:Progression-free survival analysis in BL cohort dichotomized by (A) Surgery, (B) CA19-9 levels before surgery with a cut-off of 100 U/ml and (C) type of chemotherapy received being A+G nab-paclitaxel and gemcitabine and F, FOLFIRINOX regimens. *Breslow or Log-rank test were used to test for statistical significance. **,*** in the figures indicate very significant (p<0.01) and highly significant (p<0.001) statistical differences respectively. Ns, no significant differences (p>0.05).*
