# Supplementary material for: Cytokines and Lymphoid Populations as Potential Biomarkers in Locally and Borderline Pancreatic Adenocarcinoma
Source: Cancers (Basel). 2022 Dec 5;14(23):5993. doi: 10.3390/cancers14235993 (PMC9739487; doi:10.3390/cancers14235993)
Supplement: Supplementary file 1 [file cancers-14-05993-s001.zip › supplementary/Supplementary Figure S2.pdf]

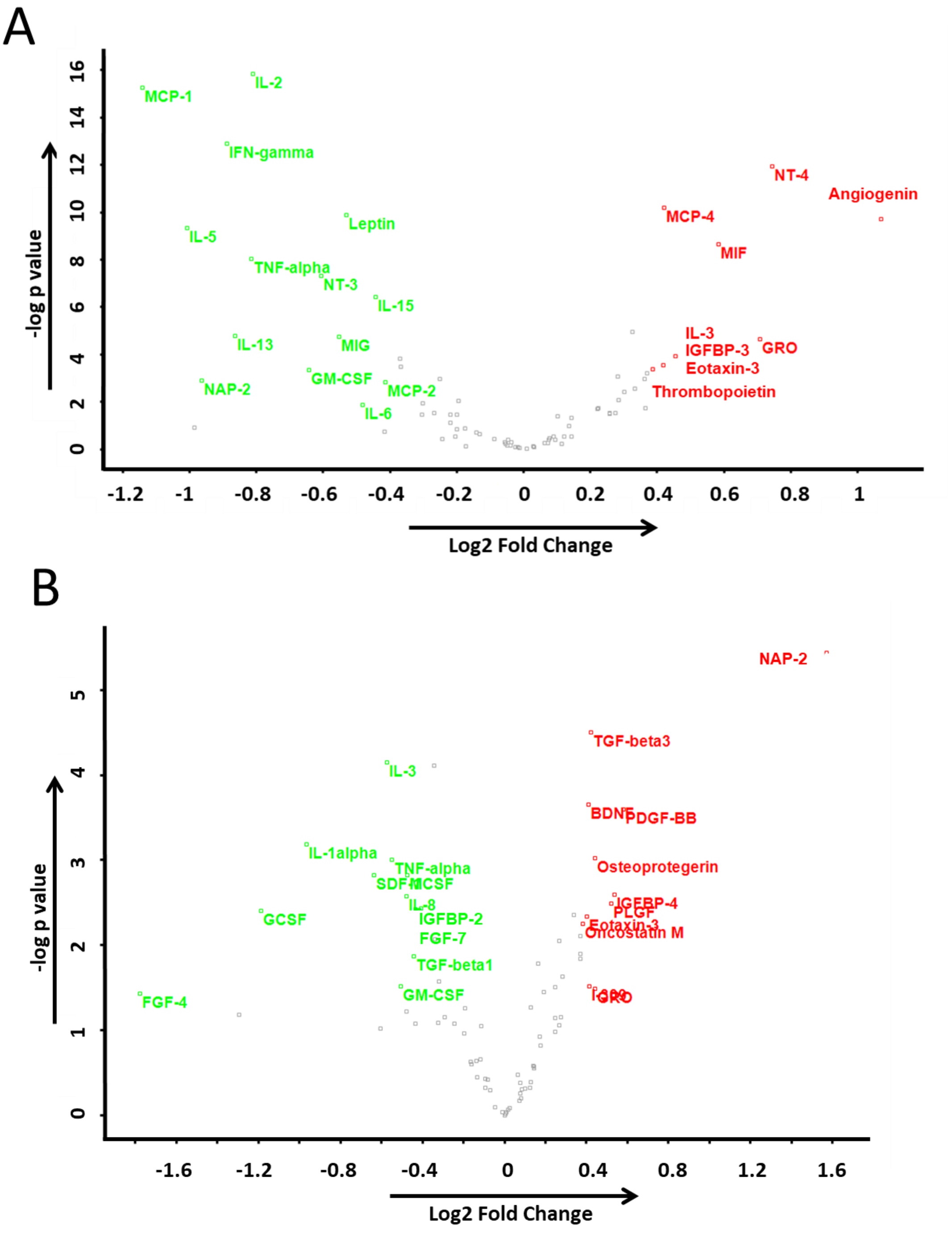

Supplementary Figure S2: Volcano plot represents the differential cytokines in baseline serum between A) patients (n=59) and healthy donors (n=22) and B) BL (n=44) and resectable patients (n=15). *Volcano plot represents the log2 fold-change of differential cytokine expression between BL and resectable patients with associated –log p-values. Green dots underexpressed proteins and red dots overexpressed proteins. Differential proteins were identified as p<0.05 and FC>30%.*
