# Supplementary material for: Cytokines and Lymphoid Populations as Potential Biomarkers in Locally and Borderline Pancreatic Adenocarcinoma
Source: Cancers (Basel). 2022 Dec 5;14(23):5993. doi: 10.3390/cancers14235993 (PMC9739487; doi:10.3390/cancers14235993)
Supplement: Supplementary file 1 [file cancers-14-05993-s001.zip › supplementary/Supplementary Figure S3.pdf]

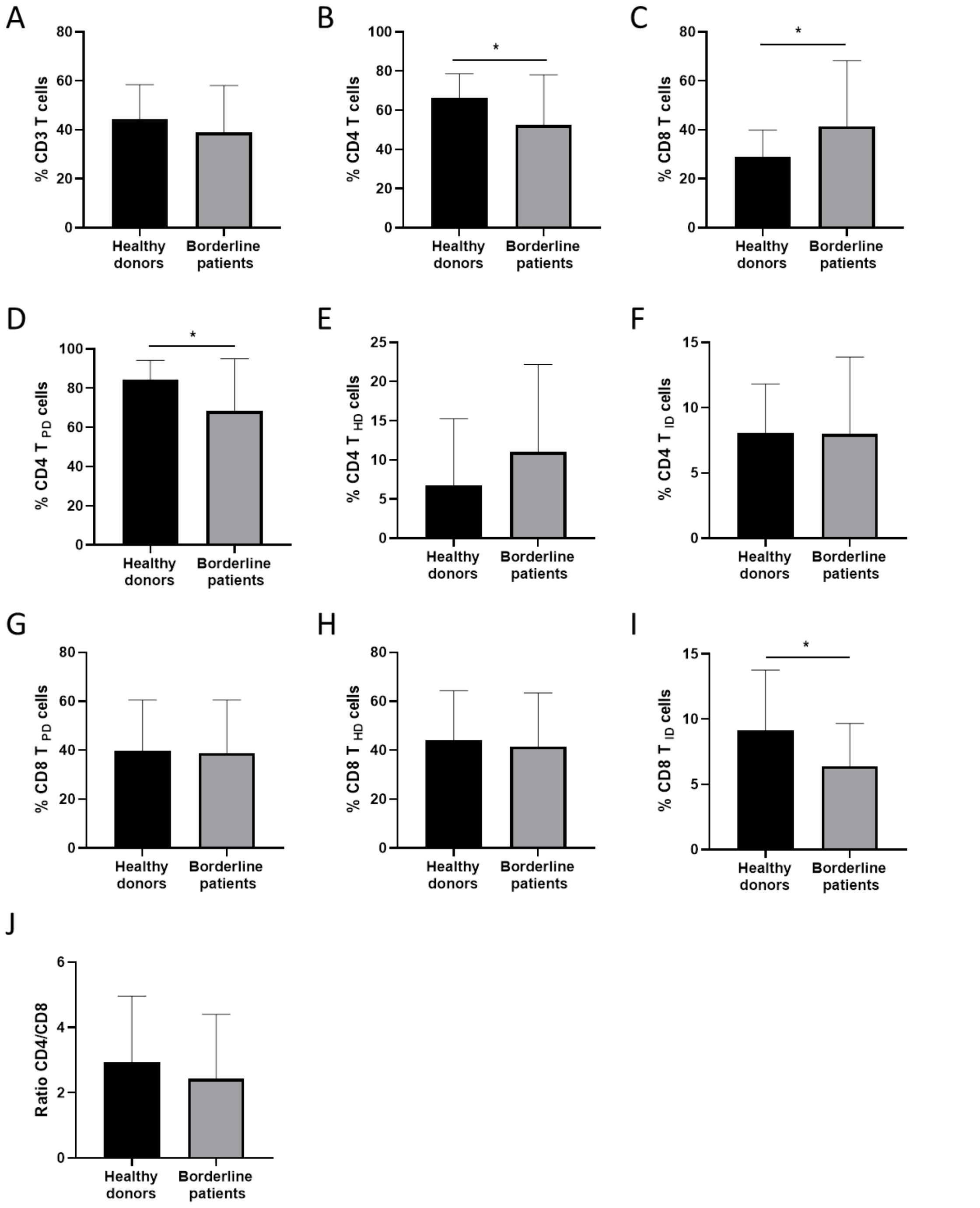

Supplementary Figure S3: Baseline profiling of CD3, CD4 and CD8 T cell populations, together with differentiation subset according to CD27/CD28 expression in healthy donors (n=22) and BL patients (n=21). *(A) $CD3^+$ cells, (B) $CD4^+$ cells, (C) $CD8^+$ cells, (D) CD4 T_{PD} cells, I CD4 T_{HD} cells, (F) CD4 T_{ID} cells, (G) CD8 T_{PD} cells, (H) CD8 T_{HD} cells, (I) CD8 T_{ID} cells, and (J) CD4/CD8 ratio are shown in the figure. Data represented as mean ± s.d. *, in the figures indicate significant ($p<0.05$).*
