# Supplementary material for: Cytokines and Lymphoid Populations as Potential Biomarkers in Locally and Borderline Pancreatic Adenocarcinoma
Source: Cancers (Basel). 2022 Dec 5;14(23):5993. doi: 10.3390/cancers14235993 (PMC9739487; doi:10.3390/cancers14235993)
Supplement: Supplementary file 1 [file cancers-14-05993-s001.zip › supplementary/Supplementary Figure S4.pdf]

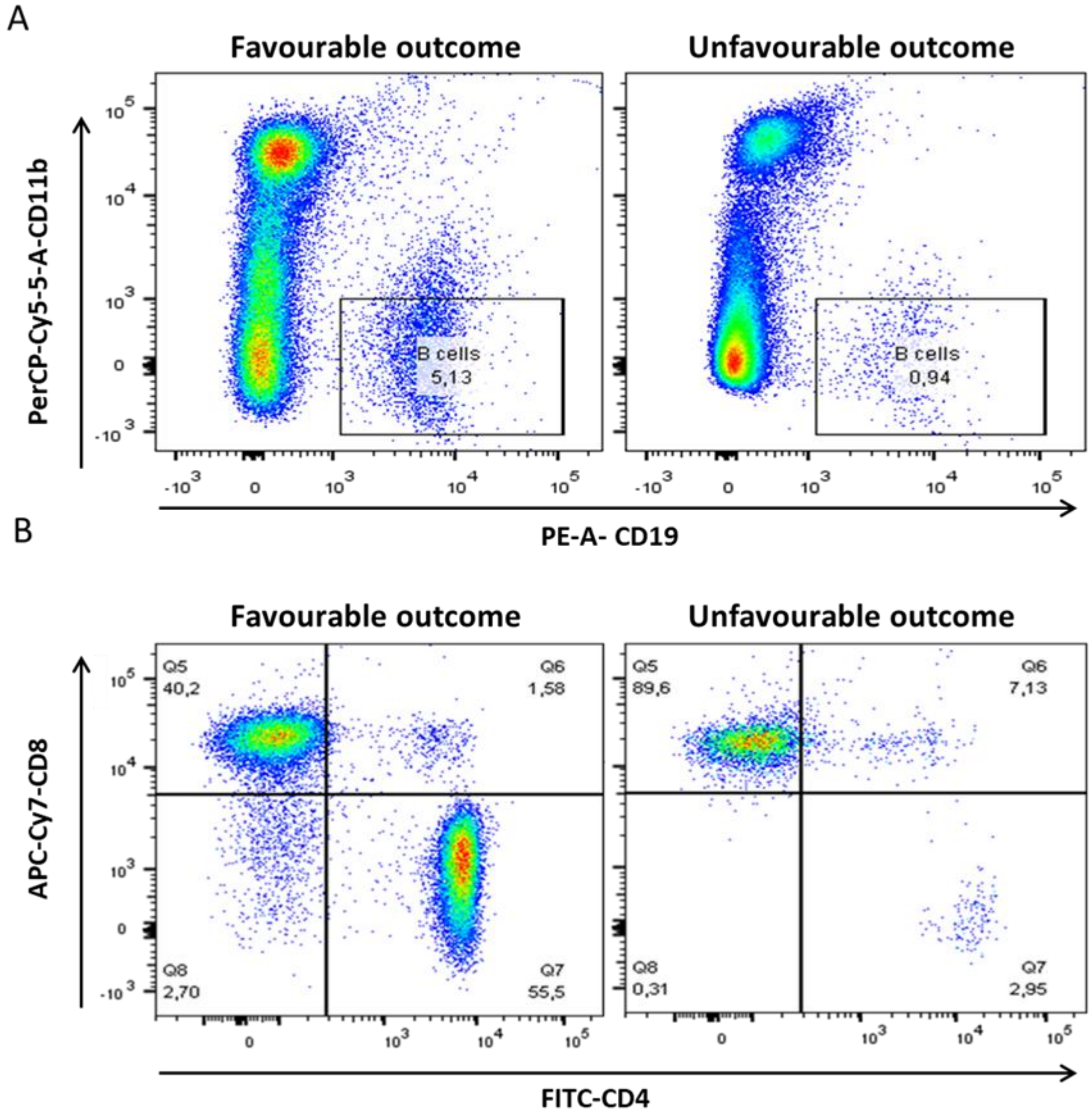

Supplementary Figure S4: Representative flow cytometry density plots showing the percentage of B cells (A) and CD4 and CD8 cells (B) at baseline comparing patients with favorable and unfavorable outcomes.
