# Supplementary material for: Cytokines and Lymphoid Populations as Potential Biomarkers in Locally and Borderline Pancreatic Adenocarcinoma
Source: Cancers (Basel). 2022 Dec 5;14(23):5993. doi: 10.3390/cancers14235993 (PMC9739487; doi:10.3390/cancers14235993)
Supplement: Supplementary file 1 [file cancers-14-05993-s001.zip › supplementary/Supplementary Figure S5new.pdf]

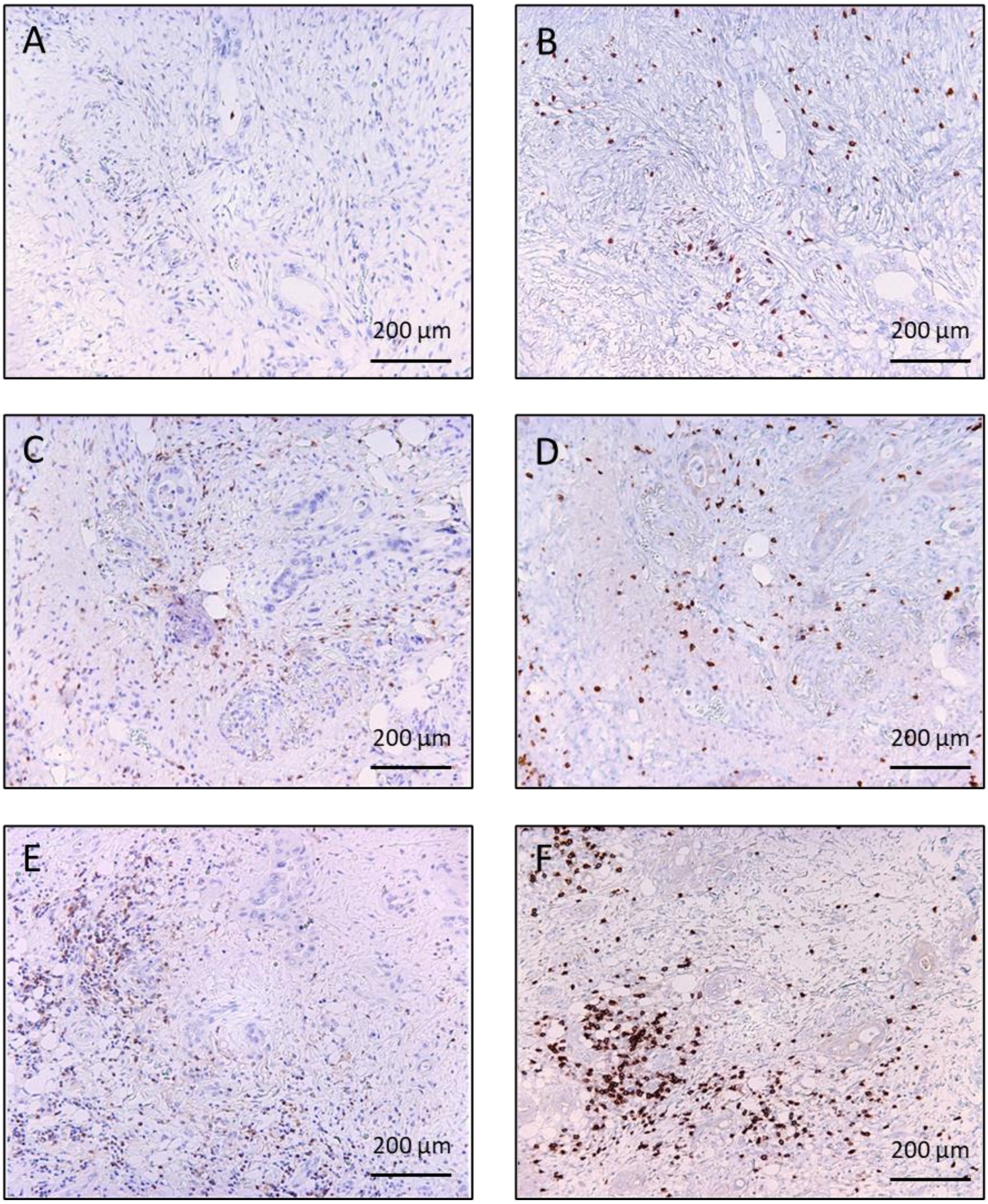

Supplementary Figure S5: Immunohistochemical staining of CD4+ T and CD8+ T cells in tumor tissue of BL cohort (n=19). *A-B represent images from patient cases with low infiltration, C-D intermediate and E-F high infiltration of lymphocytes. The images on the left represent CD4 stains and CD8 staining on the right. All images were captured with 10x objective.*
