# Supplementary material for: Cytokines and Lymphoid Populations as Potential Biomarkers in Locally and Borderline Pancreatic Adenocarcinoma
Source: Cancers (Basel). 2022 Dec 5;14(23):5993. doi: 10.3390/cancers14235993 (PMC9739487; doi:10.3390/cancers14235993)
Supplement: Supplementary file 1 [file cancers-14-05993-s001.zip › supplementary/Supplementary Table S1.pdf]

Supplementary Table S1: Antibodies used for flow cytometry and IHQ analyses

Antibody	Clone	Dilution and retrieval if needed	Reference	Company
CD11b-PerCP-Cy5	M1/70	1:52	65-0112-U100	Tonbo Biosciences (California, USA)
CD14-Violet fluor 450	61D3	1:40	75-0149-T100	Tonbo Biosciences
CD19-PE	SJ25C1	1:50	50-0198-T100	Tonbo Biosciences
CD4-FITC	M-T466	1:50	130-113-253	Miltenyi
CD27-PE	O323	1:50	50-0279-T100	Tonbo Biosciences
CD28-PE-Cy7	CD28.2	1:50	302926	BioLegend
CD3-APC	REA613	1:50	130-113-135	Miltenyi
CD8- APC-Cy7	RFT-8	1:50	A15448	Life Technologies (California, USA)
CD4	EP204	Ready-to-use pH 6, 20 min	MAD-000600QD	Vitro
CD8	SP57	Ready-to-use pH 6, 20 min	790-4460	Roche
