# Supplementary material for: Cytokines and Lymphoid Populations as Potential Biomarkers in Locally and Borderline Pancreatic Adenocarcinoma
Source: Cancers (Basel). 2022 Dec 5;14(23):5993. doi: 10.3390/cancers14235993 (PMC9739487; doi:10.3390/cancers14235993)
Supplement: Supplementary file 1 [file cancers-14-05993-s001.zip › supplementary/Supplementary Table S2.pdf]

Supplementary Table S2: KM clusters data for PFS and OS scores in BL cohort.

	Cytokine	Better (0 points (n, %))	Worse (1 point (n, %))	KM clusters
PFS	IL-10	Low (23, 52.27%)	High (21, 47.72%)	≤1 (better) vs >1 (worse) points
	MDC	Low (23, 52.27%)	High (21, 47.72%)	
	MIF	Low (24, 54.54%)	High (20, 45.45%)	
	Eotaxin-3	High (23, 52.27%)	Low (21, 47.72%)	
OS	Eotaxin-3	High (23, 52.27%)	Low (21, 47.72%)	0 (better) vs >1 (worse) points
	NT-3	High (23, 52.27%)	Low (21, 47.72%)	
	FGF-9	Low (23, 52.27%)	High (21, 47.72%)	
	IP-10	Low (21, 47.72%)	High (23, 52.27%)	
