# Supplementary material for: Cytokines and Lymphoid Populations as Potential Biomarkers in Locally and Borderline Pancreatic Adenocarcinoma
Source: Cancers (Basel). 2022 Dec 5;14(23):5993. doi: 10.3390/cancers14235993 (PMC9739487; doi:10.3390/cancers14235993)
Supplement: Supplementary file 1 [file cancers-14-05993-s001.zip › supplementary/Supplementary Table S3.pdf]

Supplementary Table S3: Baseline correlation between systemic levels of B cells, CD4/CD8 ratio and serum cytokine expression at baseline time point (n=27, 21 BL and 6 R). * p<0.05, ** p<0.01, *** p<0.001 and **** p<0.0001.

**B cells vs**	**r**	**95% confidence interval**	**R squared**	**P (two-tailed)**	**P value**
I-309	0,3912	0,01311 to 0,6714	0,153	0,0436	*
FGF-6	-0,4559	-0,7125 to -0,09175	0,2078	0,0169	*
**CD4/CD8 ratio vs**	**r**	**95% confidence interval**	**R squared**	**P (two-tailed)**	**P value**
IL-10	-0,6148	-0,8064 to -0,3064	0,378	0,0006	***
HGF	-0,485	-0,7304 to -0,1287	0,2353	0,0103	*
Eotaxin-2	-0,48	-0,7273 to -0,1223	0,2304	0,0113	*
IGFBP-1	-0,4499	-0,7087 to -0,08426	0,2024	0,0185	*
IGFBP-3	-0,3976	-0,6756 to -0,02074	0,1581	0,04	*
IGF-I	0,4114	0,03725 to 0,6844	0,1693	0,033	*
Flt-3 Ligand	0,4896	0,1347 to 0,7332	0,2397	0,0095	**
IP-10	0,515	0,1678 to 0,7485	0,2652	0,006	**
RANTES	0,6918	0,4230 to 0,8487	0,4786	<0,0001	****
